# Vanillin-Mediated Green-Synthesised Silver Nanoparticles’ Characterisation and Antimicrobial Activity: An In-Vitro Study

**DOI:** 10.7759/cureus.51659

**Published:** 2024-01-04

**Authors:** Daphane Anishya, Ravindra Kumar Jain

**Affiliations:** 1 Orthodontics and Dentofacial Orthopedics, Saveetha Dental College and Hospitals, Saveetha Institute of Medical and Technical Sciences, Saveetha University, Chennai, IND

**Keywords:** plant, dental, antimicrobial property, vanillin, white spot lesions, green synthesis, silver nanoparticle

## Abstract

Background and aim

Nanoparticles in general due to their enhanced antimicrobial effects and other beneficial effects are used in dentistry. Silver nanoparticles (AgNPs) have emerged as the metal nanoparticle with the most advantages among the many types. The objective of the study was to synthesise vanillin-mediated AgNPs, then characterise those nanoparticles and assess their antimicrobial effectiveness.

Materials and methods

One-step synthesis of stable and crystalline AgNPs was done with vanillin as the reducing and capping agent. After being crushed into powder form, the produced AgNPs were subjected to characterisation. A scanning electron Microscope SEM) analysis was done for morphological details of the AgNPs. SEM with energy dispersive X-ray spectroscopy analysis (EDAX) and Fourier transform infrared (FTIR) testing were done for elemental analysis. AgNPs' antimicrobial properties were tested using the agar well diffusion technique.

Results

The SEM analysis revealed that the synthesized AgNps were porous and agglomerative clusters and varied in sizes between 30-35 nm. SEM-EDAX revealed the presence of 76.2 weight (wt)% Ag, 4.9 wt% carbon, and 18.9 wt% of oxygen. FTIR prominent peaks were observed at 1431.97 cm and 1361.20 cm indicating the presence of AgNPs. Both low and high concentrations of AgNps showed good antimicrobial effects against *Streptococcus mutans* (*S. mutans)*.

Conclusion

Vanillin can be successfully used as a reducing agent for creating AgNPs. Due to their effective antimicrobial activity against *S.mutans* at various concentrations, vanillin-mediated AgNPs can be used with dental materials to reduce the risk of dental caries and enamel demineralization.

## Introduction

Nanoparticles are utilized in orthodontics as components of orthodontic adhesives, elastomeric ligatures, brackets, and archwires. Improved bactericidal properties, reduced friction, and increased material durability are some of the benefits. Nanoparticles applied for antimicrobial purposes in orthodontics are mainly combined with dental materials or coated on the surface of orthodontic appliances [1,2].

Among the various metal nanoparticles like titanium dioxide, zirconia dioxide, copper oxide, zinc oxide, chitosan, etc., silver nanoparticles (AgNPs) are commonly employed in scientific research and have been reported to have antimicrobial properties and biological activity against enveloped bacteria, fungi, and viruses [3-5]. Due to these qualities, Ag is frequently utilized in water purification, medicinal equipment, textile fabrics, and as dressings for burn wounds [6]. Since AgNPs have shown antimicrobial action against *Escherichia coli (E. coli), Lactobacillus Casei (L. Casei), Staphylococcus aureus (S. aureus), and Streptococcus mutans (S. mutans), *they may reduce the likelihood of enamel demineralization or white spot lesions [7]. AgNPs cause cell death by infiltrating bacterial cell walls and modifying the molecular makeup of the cell membrane, and release reactive oxygen species that inhibit DNA replication, thereby preventing biofilm formation [7,8]. Therefore, they have been included in various orthodontic products such as elastomeric modules, wires, and composites [6,7,9]. 

Diverse synthesis techniques have been used to meet the need for AgNPs. Conventional physical and chemical approaches appear to be exceedingly costly and dangerous in general. Among the several synthetic techniques for AgNPs, biological techniques appear to be the quickest, safe, and environment-friendly in ways that can generate well-defined sizes and shapes of nanoparticles [10-11].

The most popular flavour in the world, vanilla, is made from the pods of the *Vanilla planifolia *orchid [12]. Vanillin and vanillic acid have strong antibacterial properties by preventing the development of biofilms and virulence in various Gram-positive and Gram-negative pathogens, including multiple drug-resistant (MDR) bacterial strains [13]. Previously, it has been reported to synthesize noble metal nanoparticles, such as gold, for their antimicrobial characteristics [14]. Therefore, the rationale of the study was to determine whether vanillin, which was previously used to synthesize gold nanoparticles, can also be used to synthesize AgNPs. The present research was conducted to green synthesize nano‑sized particles of Ag using vanillin and study their antimicrobial properties to be used in future dental applications.

## Materials and methods

The present study was conducted in the Department of Orthodontics, Saveetha Dental College and Hospitals, Saveetha Institute of Medical And Technical Sciences (SIMATS), Chennai. Ethical approval was granted by the Institutional Ethics Committee (IHEC/SDC/ORTHO-2201/23/260), Chennai, Tamil Nadu, India.

Synthesis of vanillin-mediated AgNPs 

**Figure 1:**
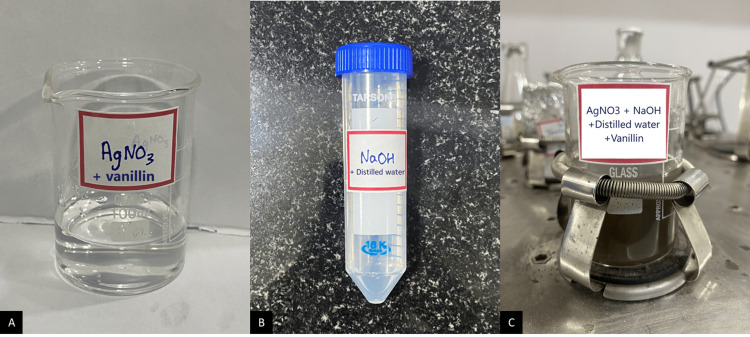
A) AgNO3 + vanillin, B) NaOH + distilled water, C) AgNO3 + vanillin + distilled water in a magnetic stirrer AgNO3: silver nitrate; NaOH: sodium hydroxide

**Figure 2:**
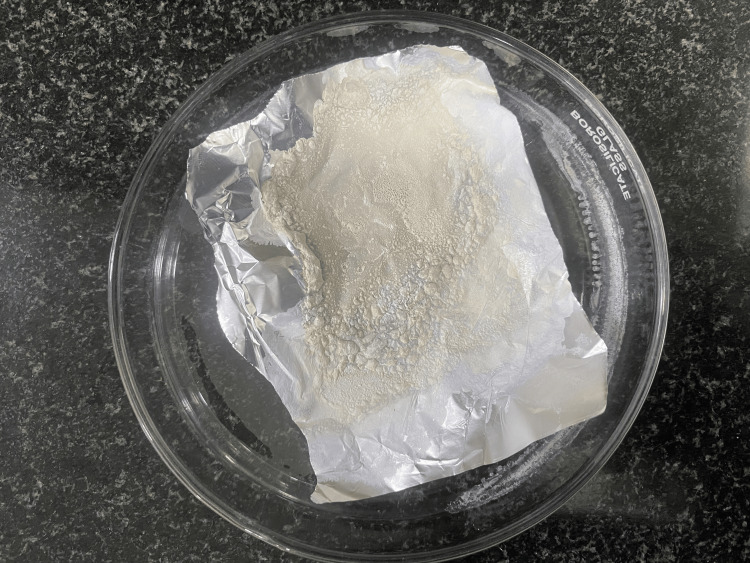
Dried substrate in powder form

Vanillin 99% pure in crystalline powder form with a molecular formula of C8H8O3 (Sisco Research Laboratories Pvt. Ltd., Mumbai, India) was used for the green synthesis. A weighing scale was used to quantify 50 mM of vanillin, which was then added to a 100 mM Ag nitrate (AgNO3) solution (Figure 1A). Distilled water was mixed with 30 mM of sodium hydroxide (NaOH) solution (Figure 1B). Both the solutions were thoroughly mixed to obtain a homogenous solution. To encourage dissolution, the preparation was kept in a magnetic stirrer and was continuously monitored to observe the visual and chemical changes that occurred during the creation of AgNPs. After one hour of nonstop magnetic stirring, the solution changed from light brown to dark brown colour (Figure 1C). The solution was left overnight in the magnetic stirrer after which it was kept in a hot air oven for drying. After 48 hours in a hot air oven, the dried substrate was extracted and ground into powder (Figure 2). The powder was sent for characterisation [14].

Characterisation of synthesised vanillin-mediated AgNPs

**Figure 3:**
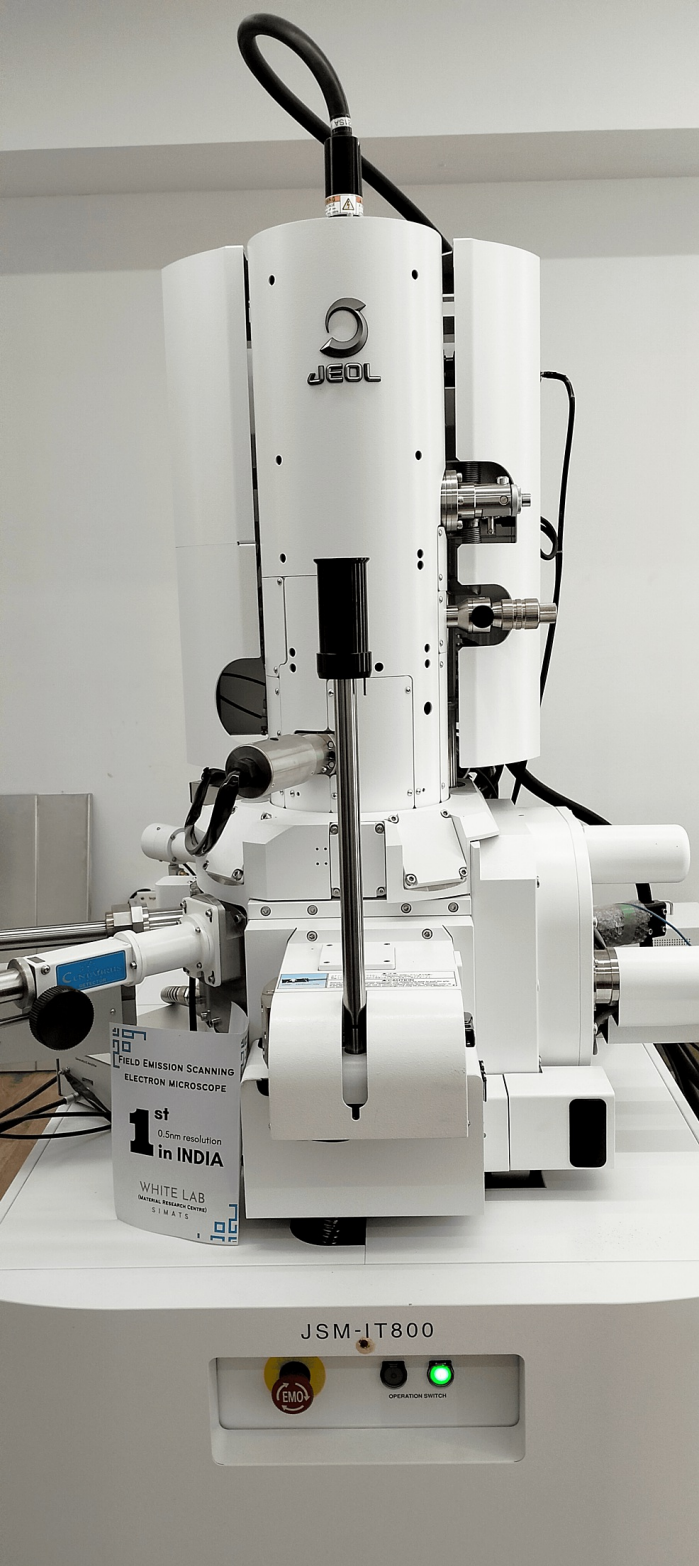
JSM-IT800 Schottky Field Emission Scanning Electron Microscope (JEOL Ltd., Tokyo, Japan)

The surface morphology of the AgNPs was investigated using a high-resolution scanning electron microscope (HR-SEM), the JSM-IT800 Schottky Field Emission Scanning Electron Microscope (JEOL Ltd., Tokyo, Japan) (Figure 3). Energy dispersive X-ray (EDX) analysis was used to determine the chemical makeup of the AgNPs. A Fourier transform infrared (FTIR) spectrophotometer was used to study the functional groups on the biaxial surface. To demonstrate how the synthesized NPs bind, FTIR is a helpful analytical technique. The Bruker Alpha II FTIR (Bruker Optics, Billerica, Massachusetts, United States) spectrometer with platinum attenuated total reflectance (ATR) was used to analyze the AgNPs in the frequency region of 4000-400 cm-1.

Antibacterial testing: agar well diffusion method

*S. mutans* is one of the most common types of bacterial species seen on the enamel surrounding the orthodontic attachments in patients undergoing fixed orthodontic treatment [15]. The antimicrobial effect against *S. mutans* was evaluated using the agar well diffusion method. Infection-causing *S.mutans* (Microbial Type Culture Collection (MTCC)-890) bacteria were stored in nutrient broth flasks that offered all the nutrients essential for inhibiting other microbial growth. A total of 150 microliters of *S. mutans *were equally distributed in wells on the nutrient agar plates under sterile conditions. Freshly formed AgNP powder was used in the wells containing *S. mutans* in two different concentrations, high concentration (HC- 100uL) and low concentration (LC- 50uL). For comparison, common antibiotics (amoxicillin) were tested against Gram-positive bacteria. The agar plate was initially incubated at 4 °C for 30 minutes for diffusion of the AgNPs into the agar medium and subsequently incubated at 37 °C for 24 hours for the bacterial growth to take place. After the incubation period, the test was considered successful if a zone of inhibition appeared around the well, indicating the presence of an antimicrobial property.

## Results

SEM

**Figure 4:**
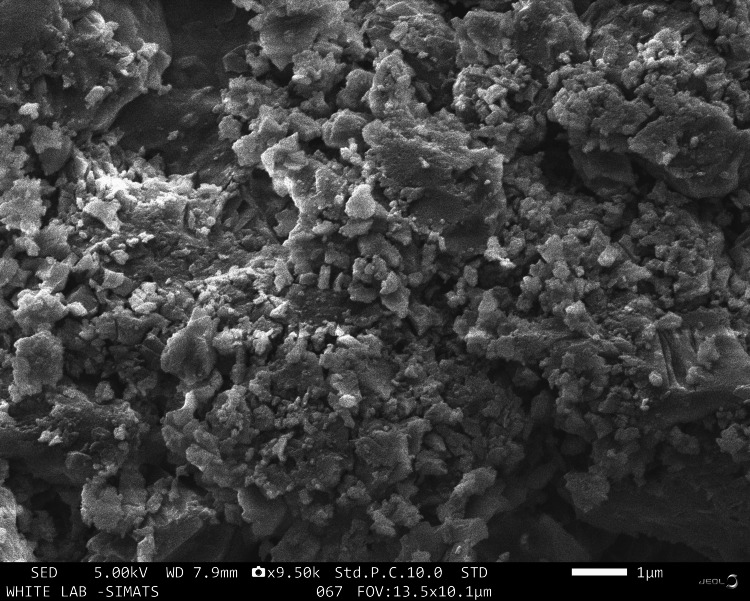
SEM Scanning electron microscopy (SEM) to evaluate the morphological details of AgNPs

The SEM was used to evaluate the morphological details such as size, shape and surface properties of the synthesised NPs. The field of view used to study the NPs was 13.5x10.1 μm. The AgNPs were visualized at a magnification of 9.50x. The AgNPs were porous and in an agglomerated cluster. The AgNPs' size was about 30-35 nm (Figure 4).

SEM-EDAX

**Figure 5:**
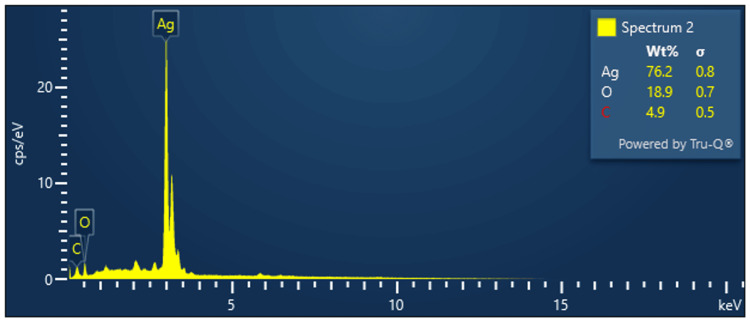
SEM-EDAX Scanning electron microscopy with energy-dispersive X-ray spectroscopy (SEM-EDAX) to evaluate the chemical/elemental composition of AgNPs

SEM-EDAX of the extracted AgNP powder was performed with an accelerating voltage of 15 KeV. It revealed the presence of 76.2 weight (wt)% Ag, 4.9 wt% carbon and 18.9 wt% of oxygen. This confirms the presence of AgNPs (Figure 5).

FTIR

**Figure 6:**
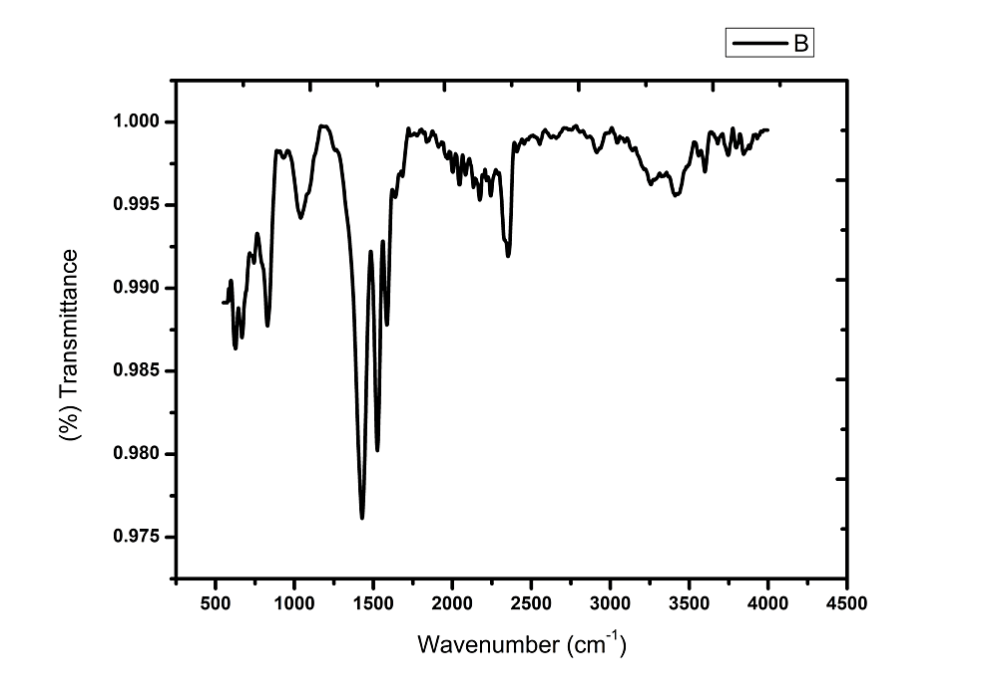
Prominent peak at 1431.97 cm-1 confirms the presence of AgNPs

The FTIR spectrum was plotted between 4,000 to 400 cm-1. The extracted powder contained AgNPs, according to the FTIR analysis. At 1431.97 cm-1 and 1361.20 cm-1, respectively, the absorption bands or strong spectral peaks were found. This confirms the presence of AgNPs (Figure 6).

Antimicrobial testing

**Figure 7:**
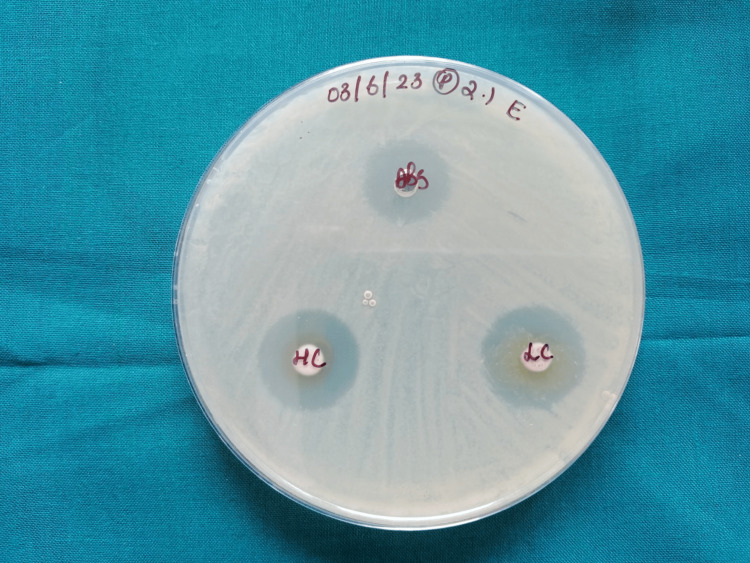
Zone of Inhibition The zone of inhibition created against *Streptococcus mutans* at different concentrations.

AgNPs' ability to resist *S. mutans* bacteria was evaluated and contrasted with amoxicillin. The zone of inhibition created against *S. mutans* provided further evidence of the prepared AgNPs' antibacterial activity. Both LC and HC of AgNPs showed a good antibacterial effect against *S.mutans *(Table 1) (Figure 7).

**Table 1:** Zone of inhibition

Microorganism	Antibiotic	50uL - low concentration	100uL - high concentration
Streptococcus mutans	16	17	19

## Discussion

Orthodontic fixed appliances hamper oral hygiene leading to plaque buildup during orthodontic treatment, further leading to the development of white spot lesions (WSLs). An NP coating of orthodontic appliances has been done in the past to lower the risk of WSLs. NPs developed chemically are highly hazardous and expensive. Recently, a variety of eco-friendly techniques have been developed to create NPs using organic and herbal ingredients. Based on previous literature, vanilla and its derivatives have antimicrobial properties [16-18]. Since it is well known that AgNPs possess potent antibacterial properties, we aimed to synthesise AgNPs utilizing vanillin as a reducing agent as vanillin has antimicrobial activity.

The objective of the current study was to synthesise AgNPs using vanillin as the reducing/capping agent and to characterise them using SEM and FTIR analysis. SEM, SEM-EDX, and FTIR data confirmed the presence of AgNPs. Antimicrobial testing was carried out once it was confirmed that the produced NPs were AgNPs. The test revealed that both at LC and HC, there was good antibacterial activity against *S. mutans*. This suggests that vanillin-mediated AgNPs possess good antibacterial properties against *S. mutans*.

A study concluded that adding 0.5% and 1% of AgNPs to orthodontic resin resulted in a significant antibacterial activity against *S. mutans* and *L. acidophilus* and helped to reduce the development of WSLs [19]. When AgNPs were incorporated into composites, they exhibited an increase in the bactericidal capabilities against both *S. mutans *and *Lactobacillus acidophilus* (*L. acidophilus*). AgNPs could potentially be included in commercial orthodontic adhesives to boost their bactericidal activity without changing their mechanical characteristics [20]. An in-vitro investigation was done to evaluate the anti-adherent and antibacterial activity of orthodontic brackets with AgNP coating on their surfaces. They came to the conclusion that the Ag coating has antibacterial characteristics since it prevented *S. mutans* and *Streptococcus sobrinus* (*S. sobrinus*) from adhering to the orthodontic brackets [21]. The results of these studies suggest that AgNps can be used in numerous orthodontic appliances and work efficiently against *S. mutans*.

According to a study by Arya et al., vanillin can be used as a reducing/capping agent to create stable, crystalline gold NPs [14]. In the present study, vanillin-mediated AgNPs were synthesised. In a study by Thaweboon et al., 0.5% vanillin was added to self-curing polymethyl methacrylate (PMMA) resin to make orthodontic retainers. This significantly reduced the ability of cariogenic bacteria (*S. mutans, S. sobrinus, L. casei, and L. acidophilus)* to form biofilms, hence lowering the incidence of WSLs [17]. A similar study by Thaweboon et al. reported that in comparison to samples without vanillin, all samples incorporating vanillin (0.5%, 1%, and 5%) showed a significant inhibitory effect against* L. casei* and *S. mutans* biofilm [18]. As stated in a study by Qassab-Bashi, the mean value of optical density for both bacteria significantly decreased when vanillin (2%) was used alone or in combination with dentifrice when compared to the control reading. This indicates that it works well to prevent enamel demineralisation by limiting the growth of cariogenic microorganisms [22].

Many clinical investigations have been conducted recently to support the application of AgNPs to prevent demineralization of enamel surfaces. In split-mouth research involving 40 children, Salas-Lopez et al. randomly assigned either a traditional pit and fissure sealant or a sealant containing AgNPs on erupted permanent first molars. In comparison to the usual sealant, he claimed that the AgNP-mixed sealant greatly reduced tooth demineralization as well as boosted remineralization [23]. In a double-blind prospective randomised clinical experiment conducted by Ali et al., patients received mouthwash that was either chlorhexidine (CHX), fluoride, or nanosilver, at random. The three groups formed WSLs at very different rates, according to their reports. When it involved minimizing WSLs during orthodontic treatment, nanosilver mouthwash was considered to be superior to CHX and fluoride mouthwash [24].

Similar to the earlier investigations, the current work produced AgNPs using vanillin as the reducing/capping agent had good antibacterial properties against *S. mutans*. As a result, they can be used in conjunction with orthodontic appliances to minimize the development of biofilm. Considering the results of the present study's antimicrobial properties, it would be beneficial to incorporate both concentrations of AgNPs in orthodontic adhesives in order to prevent WSLs in patients undergoing orthodontic treatment.

Vanilla is inexpensive and widely accessible. AgNPs were made in a remarkably minimal amount of time using green synthesis. Additionally, the waste or extra material left after AgNP production was not harmful, making this procedure ecologically friendly.

Limitations

The potential hazards associated with nanotechnology are now greater. NPs' small size makes them risky for inhalation, and they may trigger a number of fatal diseases when inhaled for just 60 seconds. The study's failure to examine NP coating stability is still another drawback. Occasionally, NPs may cause unanticipated issues. The study's focus on the antibacterial properties of a single bacteria, *S. mutans*, is another drawback. Additional microbes included in the testing would increase the validity of the antimicrobial activity of the nanomaterial.

## Conclusions

We may conclude from the current study's results that vanillin can be employed to create AgNPs. When used against *S mutans*, the produced AgNPs exhibit significant antibacterial properties. Furthermore, vanillin itself possesses certain antibacterial qualities that might be helpful in conjunction with orthodontic appliances like wires, brackets, and adhesives to prevent plaque buildup and WSLs in patients undergoing fixed orthodontic treatment.
